# The phosphatase activity of soluble epoxide hydrolase regulates ATP‐binding cassette transporter‐A1‐dependent cholesterol efflux

**DOI:** 10.1111/jcmm.14519

**Published:** 2019-08-22

**Authors:** Chih‐Chan Lien, Chia‐Hui Chen, Yeng‐Ming Lee, Bei‐Chia Guo, Li‐Ching Cheng, Ching‐Chien Pan, Song‐Kun Shyue, Tzong‐Shyuan Lee

**Affiliations:** ^1^ Graduate Institute and Department of Physiology, College of Medicine National Taiwan University Taipei Taiwan; ^2^ Department of Physiology, School of Medicine National Yang‐Ming University Taipei Taiwan; ^3^ Institute of Biomedical Sciences Academia Sinica Taipei Taiwan; ^4^ Graduate Institute of Life Science National Defense Medical Center Taipei Taiwan; ^5^ Division of Basic Medical Sciences, Department of Nursing Chang Gung University of Science and Technology Taoyuan Taiwan

**Keywords:** ABCA1, macrophage foam cell, phosphatase, soluble epoxide hydrolase

## Abstract

The contribution of soluble epoxide hydrolase (sEH) to atherosclerosis has been well defined. However, less is understood about the role of sEH and its underlying mechanism in the cholesterol metabolism of macrophages. The expression of sEH protein was increased in atherosclerotic aortas of apolipoprotein E‐deficient mice, primarily in macrophage foam cells. Oxidized low‐density lipoprotein (oxLDL) increased sEH expression in macrophages. Genetic deletion of sEH (*sEH* ^−/−^) in macrophages markedly exacerbated oxLDL‐induced lipid accumulation and decreased the expression of ATP‐binding cassette transporters‐A1 (ABCA1) and apolipoprotein AI‐dependent cholesterol efflux following oxLDL treatment. The down‐regulation of ABCA1 in *sEH* ^−/−^ macrophages was due to an increase in the turnover rate of ABCA1 protein but not in mRNA transcription. Inhibition of phosphatase activity, but not hydrolase activity, of sEH decreased ABCA1 expression and cholesterol efflux following oxLDL challenge, which resulted in increased cholesterol accumulation. Additionally, oxLDL increased the phosphatase activity, promoted the sEH‐ABCA1 complex formation and decreased the phosphorylated level of ABCA1 at threonine residues. Overexpression of phosphatase domain of sEH abrogated the oxLDL‐induced ABCA1 phosphorylation and further increased ABCA1 expression and cholesterol efflux, leading to the attenuation of oxLDL‐induced cholesterol accumulation. Our findings suggest that the phosphatase domain of sEH plays a crucial role in the cholesterol metabolism of macrophages.

## 1 INTRODUCTION

Soluble epoxide hydrolase (sEH), an enzyme with COOH‐terminal epoxide hydrolase (EH) and NH_2_‐terminal lipid phosphatase (PT) activities, is widely distributed in mammalian tissues and plays an important role in regulating multiple physiological functions.^1^, ^2^ The implication of hydrolase activity of sEH in the metabolism of epoxyeicosatrienoic acids (EETs), inflammation and hypertension has been well documented.^3^, ^4^, ^5^, ^6^ For example, pharmacological inhibition of the EH activity of sEH or deletion of the sEH gene causes an increase in the accumulation of EETs and leads to the attenuation of angiotensin II–induced hypertension and cardiac hypertrophy and lipopolysaccharide‐induced inflammation in vitro and in vivo.^5^, ^6^, ^7^, ^8^ Oral administration with inhibitors targeting EH activity of sEH or genetic disruption of sEH significantly retards the progression of atherosclerosis in hyperlipidemic mouse models.^9^, ^10^ Multiple lines of evidence demonstrate that the PT domain of sEH also has a crucial role in the regulation of cholesterol metabolism and cell growth in hepatocytes.^11^, ^12^ Moreover, the sEH Glu287Arg mutant, which has reduced PT activity, is known to be a risk factor for the development of coronary artery disease (CAD).^13^, ^14^ Additionally, this sEH polymorphism is closely associated with the increase in plasma cholesterol and triglyceride in familial hypercholesterolaemia patients.^15^ However, the role of the PT activity of sEH in the cholesterol metabolism of macrophage foam cells remains to be investigated.

Atherosclerosis is a chronic inflammatory process, caused by the deregulation of lipid metabolism of macrophages within arterial walls, ultimately leading to the clinical complications of CADs in humans.^16^ Although the detailed mechanisms of this disease are not yet fully defined, it has been believed that regulation of cholesterol metabolism and the inflammatory response by lipid‐laden macrophages are critical steps in the initiation and progression of atherosclerosis.^17^, ^18^ Several lines of evidence suggest that inhibition of foam cell formation retards the progression of atherosclerosis in experimental animal models.^19^, ^20^, ^21^ The formation of foam cells is primarily caused by uncontrolled uptake of oxidized low‐density lipoprotein (oxLDL) or impaired cholesterol efflux in macrophages, which result in excessive oxLDL‐derived lipid accumulation inside macrophages.^19^, ^22^ Scavenger receptors (SRs) class A SR (SR‐A) and CD36 are responsible for internalization of oxLDL.^23^, ^24^ By contrast, the efflux of accumulated cholesterol in macrophages is mediated through reverse cholesterol transporters (RCTs) including SR‐BI and the ATP‐binding cassette transporters A1 and G1 (ABCA1 and ABCG1).^25^, ^26^ Therefore, the lipid content of foam cells is dynamically regulated by these SRs and cholesterol efflux transporters. However, less is known about the interplay between sEH and foam cells. To this end, further investigation delineating the expression and the mechanisms of sEH on the formation of foam cells is warranted.

Given the importance of sEH in the development of CADs, in this study we investigated the role and the mechanism of sEH in regulating the cholesterol metabolism of macrophage foam cells. We first investigated the expression and distribution of sEH in atherosclerotic lesions of apolipoprotein E null (*Apoe* ^−/−^) mice. Our second aim was to characterize the role of sEH in oxLDL‐deregulated cholesterol metabolism and the underlying molecular mechanism in bone marrow‐derived macrophage (BMDM) foam cells by use of loss‐of‐function and gain‐of‐function strategies. Here, we report that the PT activity, but not the EH activity, of sEH plays a crucial role in regulating ABCA1‐dependent cholesterol efflux during the transformation of macrophage foam cells. Our findings provide a novel explanation for the anti‐atherogenic properties of sEH and suggest a molecular target for the treatment of atherosclerosis.

## 2 MATERIALS AND METHODS

### 2.1 Reagents

Human LDL, 4‐nitrophenyl phosphate, ebselen, mouse antibody for α‐tubulin, and phosphatase inhibitor cocktails 1 and 2 were from Sigma‐Aldrich. Rabbit antibodies for CD36, sEH and phospho‐threonine (p‐Thr), and goat antibodies for SR‐A and ABCG1 were from Santa Cruz Biotechnology. Mouse antibodies for ABCA1, rabbit antibodies for SR‐BI and F4/80 were from Abcam. 12‐(3‐adamantan‐1‐yl‐ureido)‐dodecanoic acid (AUDA), NBD cholesterol, oleic acid (OA) and TBARS assay kits were from Cayman Chemical. Cholesterol and triglyceride assay kits were from Randox. Peroxidase‐conjugated antimouse IgG, anti‐rabbit IgG, anti‐goat IgG and anti‐rat IgG antibodies were from Jackson ImmunoResearch.

### 2.2 Animals

All animal experiments were approved by the Animal Care and Utilization Committee of National Yang‐Ming University. C57BL/6 mice were purchased from the National Laboratory Animal Center, National Science Council, and *Apoe* ^−/−^ mice and *EPXH2* ^−/−^ sEH‐deficient (sEH KO) mice were obtained from the Jackson Laboratory. Mice were housed in barrier facilities on a 12‐hour light/12‐hour dark cycle and fed with regular chow (4.5% fat by weight, 0.02% cholesterol; Newco Distributors).

### 2.3 Immunohistochemistry

Formalin‐fixed, paraffin‐embedded tissue blocks were cut into 8 µM sections. Sections were deparaffinized, rehydrated and covered with 3% H_2_O_2_ for 10 minutes. After blocking with BSA, slides were incubated with primary antibodies for 1 hour at 37°C and with corresponding secondary antibodies for an additional 1 hour. Antigenic sites were visualized by the addition of DAB. Slides were counterstained with haematoxylin.

### 2.4 Cell culture

Bone marrow‐derived macrophages were prepared as previously described.^27^ Briefly, mice were killed by CO_2_ inhalation and mononuclear cells from their femurs were harvested by Percoll (1.073 g/cm^3^) density‐gradient centrifugation. The cells were then seeded in MEMα supplemented with 50 ng/mL macrophage colony‐stimulating factor, 10% FBS and penicillin (100 U/mL)/streptomycin (100 μg/mL) for differentiation for 5 days at 37°C. The differentiated macrophages were then subjected to further experiments. HEK293 cells and Huh7 hepatoma cells were cultured in DMEM supplemented with 10% FBS and penicillin (100 U/mL)/streptomycin (100 µg/mL) at 37°C.

### 2.5 Low‐density lipoprotein modification

The oxLDL was prepared as described previously.^28^ LDL was exposed to 5 µM CuSO_4_ for 24 hours at 37°C, and Cu^2+^ was then removed by extensive dialysis. The extent of modification was determined by measuring thiobarbituric acid‐reactive substances (TBARs). OxLDL containing approximately 30‐60 nmol of TBARs, defined as malondialdehyde equivalents per mg of LDL protein, was used for experiments.

### 2.6 PT activity assay

Bone marrow‐derived macrophages with or without indicated treatments were collected in phosphate‐buffered saline (PBS), sonicated, and the supernatant was collected by centrifugation at 10 000 *g* for 10 minutes. This cell lysate was added to 4‐nitrophenyl phosphate to 2 mM and incubated at 37°C for 1 hour. The yellow colour product was detected by OD_405_ nm to determine PT activity.

### 2.7 Oil red O staining

Cells were fixed with 4% paraformaldehyde and then stained by 0.5% oil red O. Haematoxylin was used as a counterstain. The intracellular lipid content was evaluated by alcohol extraction after oil red O staining. The absorbance at 540 nm was measured using a microplate reader (BioTek Instruments).

### 2.8 Cholesterol and triglyceride measurement

Cellular cholesterol and triglyceride were extracted by hexane/isopropanol (3/2, v/v). After removing cellular debris, the supernatant was dried under nitrogen. The levels of cholesterol and triglyceride were measured using cholesterol and triglyceride assay kits (Randox).

### 2.9 Immunoprecipitation assay and Western blot analysis

The methods for immunoprecipitation and Western blot analysis as previously described.^29^ Cells were rinsed with phosphate‐buffered saline (PBS) and then lysed in immunoprecipitation lysis buffer (50 mM Tris pH 7.5, 5 mM EDTA, 300 mM NaCl, 1% Triton X‐100, 1 mM phenylmethylsulfonyl fluoride, 10 µg/mL leupeptin and 10 µg/mL aprotinin). Aliquots (1000 µg) of cell lysates were incubated with specific primary antibody overnight and then with protein A/G‐Sepharose for 2 hours. Immune complexes were collected by centrifugation at 5000 *g* for 10 minutes, washed 3 times with cold PBS and then eluted in sodium dodecyl sulphate (SDS) lysis buffer (1% Triton, 0.1% SDS, 0.2% sodium azide, 0.5% sodium deoxycholate, 10 µg/mL leupeptin and 10 µg/mL aprotinin). Eluted protein samples were separated by 8% or 10% SDS‐PAGE. After transfer to membranes, the samples were incubated with primary antibodies, washed and then incubated with secondary antibodies conjugated with horseradish peroxidase. Bands were revealed using an enzyme‐linked chemiluminescence detection kit (PerkinElmer), and signals were quantified using Imagequant 5.2 software (Healthcare Bio‐Sciences).

### 2.10 Cholesterol efflux assay

Macrophages were equilibrated with NBD cholesterol (1 µg/mL) for 12 hours. These cells were washed with PBS and incubated with oxLDL (50 µg/mL) in RPMI 1640 medium for another 12 hours in the presence of apoAI (10 µg/mL) or HDL (50 µg/mL). The fluorescence‐labelled cholesterol released from the cells into the medium was analysed using a multilabel counter (PerkinElmer) with 485 nm excitation and 535 nm emission.

### 2.11 Preparation of EH and PT domains of sEH expression vectors and adenovirus

C‐terminal hydrolase and N‐terminal phosphatase domains of sEH were isolated by polymerase chain reaction (PCR) with different primer sets from a human sEH cDNA clone. PCR was performed with Hi Fi *Taq* DNA polymerase (Geneaid) as follows: 2 minutes at 94°C, 15 seconds at 94°C, 30 seconds at 58°C and 1 minutes at 72°C for 35 cycles. The primers for full‐length sEH were 5′‐TTACGCGTGCGCTGCGTGTAGCCG‐3′ (forward primer; underline, MluI site) and 5′‐GGTCTAGACTAAATCTTGGAGGTCACTG‐3′ (reverse primer; underline, XbaI site). The primers for the N‐terminal PT domain were 5′‐TTACGCGTGCGCTGCGTGTAGCCG‐3′ (forward with MluI site) and 5′‐GGTCTAGACTACCCTGTGACCTTCTCCA‐3′ (reverse with XbaI site). The primers for the C‐terminal EH domain were 5′‐TTACGCGTGTCAGCCATGGATATGTGAC‐3′ and 5′‐GGTCTAGACTAAATCTTGGAGGTCACTG‐3′. PCR products were cloned into the pGEM vector according to the manufacturer's instructions (Promega). Clones were confirmed by sequencing. These three plasmids were digested with MluI and XbaI, and inserts were ligated into the pCMV5 N‐Flag vector. A replication‐defective recombinant adenoviral vector containing a human phosphoglycerate kinase (hPGK) promoter driving the human EH domain (Adv‐EH) or PT domain (Adv‐PT) of sEH, as well as hPGK alone to serve as a control (Adv‐null), was constructed. Recombinant adenovirus was generated by homologous recombination, amplified in HEK293 cells, purified by CsCl ultracentrifugation, and stored in 10 mm Tris‐HCl (pH 7.4), 1 mm MgCl_2_ and 10% (vol/vol) glycerol in liquid nitrogen until used for experiments. The titres of adenovirus were determined by plaque assay in HEK293 cells. Macrophages were infected with 50 MOI of adenovirus for 24 hours and then subjected to experiments.

### 2.12 Statistical analysis

Data are presented as mean ± SEM from 5 mice or 5 independent cell experiments. Data from animal studies were evaluated by parametric tests. One‐way ANOVA followed by the LSD test was used for multiple comparisons. Data from cell studies were evaluated by non‐parametric tests. The Mann‐Whitney *U* test was used to compare 2 independent groups. The Kruskal‐Wallis followed by Bonferroni post hoc tests was used to account for multiple comparisons. SPSS v 20.0 (SPSS Inc) was used for analysis. Differences were considered statistically significant at *P* < 0.05.

## 3 RESULTS

### 3.1 **Expression of sEH is increased in atherosclerotic lesions of *Apoe*** ^−^ **^/^** ^−^ **mice**

**Figure 1:**
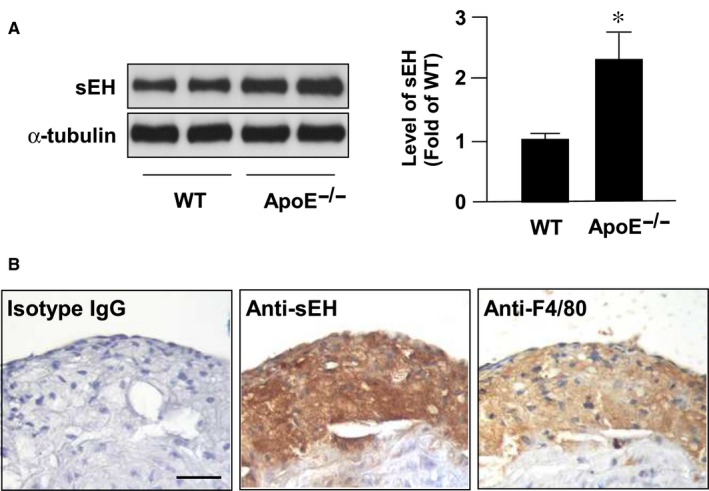
sEH expression is increased in atherosclerotic lesions. Aortas were collected from 5‐ month‐old wild type (WT) mice and apolipoprotein E knockout (Apoe‐/‐) mice. (A) Western blot analysis of sEH and a‐tubulin. Data are mean ± SD from 5 mice. *P < 0.05 vs. WT mice. (B) Immunostaining of macrophage foam cells with (left) control normal rabbit IgG, (center) anti‐sEH, and (right) anti‐F4/80 antibody. Cell nuclei were stained with hematoxylin. Bar = 50 μm

We first measured the expression of sEH in normal and atherosclerotic aortas from WT mice and *Apoe* ^−/−^ mice. Western blot analyses demonstrated that the expression of sEH was increased in the aortas of *Apoe* ^−/−^ mice relative to those of control WT mice (Figure 1A). In addition, sEH was predominantly expressed in macrophage foam cells as revealed by immunohistochemistry (Figure 1B).

### 3.2 Genetic deletion of sEH amplifies oxLDL‐induced lipid accumulation in macrophages

**Figure 2:**
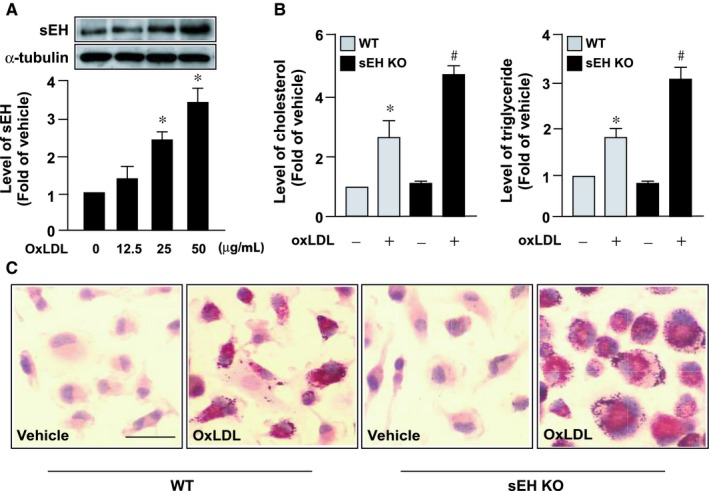
Genetic ablation of sEH exacerbates oxLDL‐induced cholesterol accumulation in macrophages. A, Western blot analysis of sEH protein expression in bone marrow‐derived macrophages (BMDMs) treated with indicated concentrations of oxidized low‐density lipoprotein (oxLDL) for 24 h. B, Wild‐type (WT) or *EPXH2* ^−/−^ sEH‐deficient (sEH KO) BMDMs were treated with or without 50 µg/mL oxLDL for 24 h. B, Measurements of intracellular levels of cholesterol and triglycerides. C, Cells were fixed and subjected to oil red O staining. Cellular nuclei were stained with haematoxylin. Bar = 20 µm. Data are mean ± SD from 5 independent experiments. *P < 0.05 vs vehicle‐treated control, #, *P* < 0.05 vs oxLDL‐treated WT macrophages

We found that oxLDL, the most important atherogenic factor, up‐regulated the expression of sEH protein (Figure 2A). Thus, we have been suggested that sEH is involved in the regulation of lipid metabolism of macrophage foam cells. As shown in Figure 2B,C, oxLDL‐induced lipid accumulation was significantly higher in *EPXH2* ^−/−^ sEH‐deficient macrophages than in WT cells, as evidenced by an increase in intracellular levels of cholesterol and triglycerides.

### 3.3 Deficiency of sEH impairs cholesterol efflux by down‐regulating ABCA1 expression following oxLDL challenge

**Figure 3:**
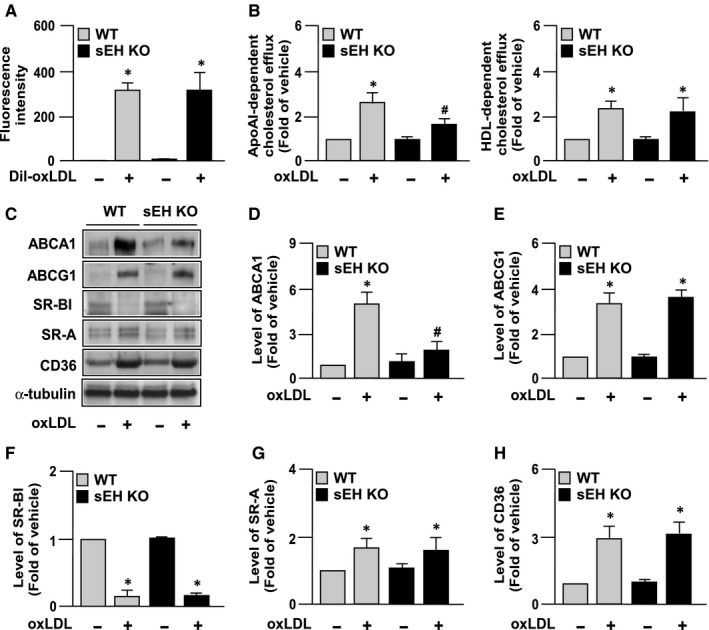
Genetic deletion of sEH impairs cholesterol efflux and down‐regulates expression of ATP‐binding cassette transporter A1 following oxLDL treatment in macrophages. A, For Dil‐oxLDL binding assays, WT or *EPXH2* ^−/−^ sEH‐deficient (sEH KO) macrophages were treated with or without Dil‐oxLDL (10 µg/mL) for 4 h at 4°C. Cellular lysates were analysed by fluorometry. B, Macrophages were treated with NBD cholesterol (1 µg/mL) for 12 h, followed by oxLDL (50 µg/mL) in the presence of apoAI (10 µg/mL) or HDL (50 µg/mL) for an additional 12 h. Cholesterol efflux was quantified as a percentage of fluorescence in the medium relative to the total amount of fluorescence. C‐H, BMDMs were incubated with 50 µg/mL oxLDL for 24 h and cell lysates were subjected to (C) Western blotting. Blots were quantitated to determine the protein levels of (D) ABCA1, (E) ABCG1, (F) SR‐BI, (G) SR‐A, (H) CD36, with α‐tubulin as a control for normalization. Fold induction was defined as level of protein relative to the untreated group set as 1. Data shown are mean ± SD from 5 independent experiments. *P < 0.05 vs untreated groups, #; *P* < 0.05 vs oxLDL‐treated WT macrophages

The cellular cholesterol content of macrophage foam cells in atherosclerotic lesions is dynamically regulated by SR‐mediated oxLDL internalization and RCT‐mediated cholesterol efflux.^23^, ^24^ We therefore delineated the role of sEH in regulating expression of SRs and RCTs and cholesterol metabolism in macrophages. We demonstrated that genetic deletion of sEH did not affect oxLDL binding and HDL‐dependent cholesterol efflux, but reduced apoAI‐dependent cholesterol efflux (Figure 3A,B). Moreover, deficiency of sEH decreased oxLDL‐up‐regulated ABCA1 expression; however, it had no effect on the oxLDL‐mediated regulation of expression of ABCG1, SR‐BI SR‐A and CD36 (Figure 3C‐H). Thus, sEH is involved in the regulation of cholesterol homeostasis in response to oxLDL stimuli in macrophages.

### 3.4 Deficiency of sEH promotes the turnover of ABCA1 protein

**Figure 4:**
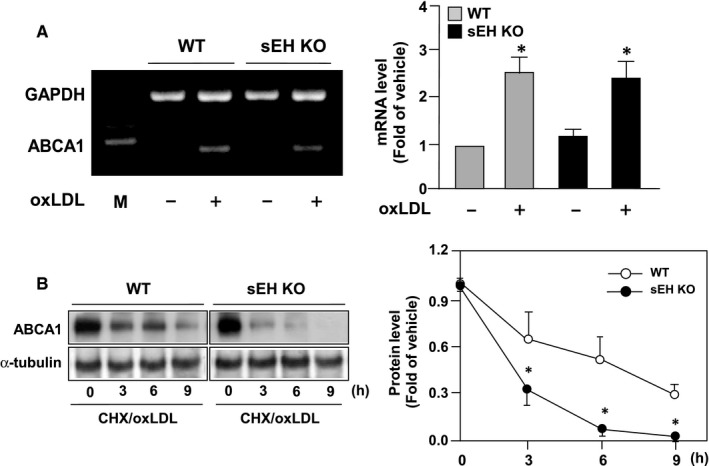
Genetic disruption of sEH promotes the degradation of ABCA1 protein in oxLDL‐treated macrophages. A, Total RNA was isolated at 6 h after oxLDL incubation and ABCA1 and GAPDH mRNA was determined by semi‐quantitative RT‐PCR analysis. B, Macrophages were incubated in the presence of 2 µg/mL cycloheximide (CHX) to inhibit protein translation with oxLDL (50 µg/mL) for the time periods indicated. Data shown are mean ± SD from 5 independent experiments. *P < 0.05 vs oxLDL‐treated WT macrophages

We further delineated the molecular mechanisms underlying the effect of sEH deficiency on the down‐regulation of ABCA1 by measuring the gene expression and protein stability of ABCA1 in the presence of oxLDL. Our results showed that loss of function of sEH did not affect oxLDL‐induced ABCA1 gene expression (Figure 4A); however, the degradation rate of ABCA1 protein was promoted by oxLDL treatment (Figure 4B). These results suggest that sEH may play a key role in the stability of ABCA1 protein in response to oxLDL challenge.

### 3.5 PT activity of sEH regulates ABCA1 stability and cholesterol metabolism in oxLDL‐treated macrophages

**Figure 5:**
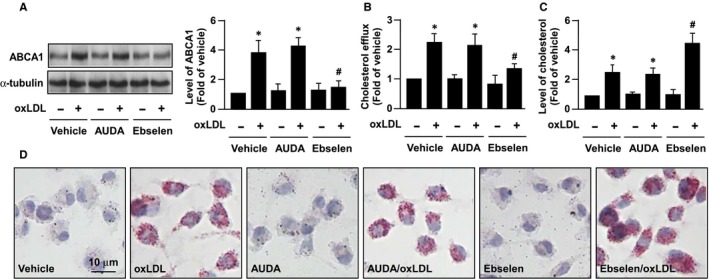
Role of sEH phosphatase activity in ABCA1 expression and phosphorylation in oxLDL‐treated macrophages. A and B, Macrophages were pre‐treated with 10 µM AUDA (an inhibitor for hydrolase activity of sEH) or ebselen (an inhibitor for phosphatase activity of sEH) for 2 h and then oxLDL (50 µg/mL) for additional 24 h. A, Western blot analysis of ABCA1 and α‐tubulin. B, Cholesterol efflux was measured in the presence of apoAI. C and D, Intracellular cholesterol and lipid accumulation were evaluated by the colorimetric assay kit and oil red O staining. Data are mean ± SD from 5 independent experiments. *P < 0.05 vs vehicle group, # *P* < 0.05 vs oxLDL‐only group

We next investigated whether the PT activity or the EH activity of sEH participates in regulating expression of ABCA1 upon oxLDL treatment. Bone marrow‐derived macrophages were pre‐treated with AUDA (an inhibitor of EH activity of sEH) or ebselen (an inhibitor of sEH PT activity). As shown in Figure 5A‐D, treatment with ebselen attenuated the oxLDL‐induced increase in the protein expression of ABCA1, apoAI‐dependent cholesterol efflux and cholesterol accumulation in macrophages. However, AUDA treatment failed to produce such effects, suggesting that the PT activity of sEH is crucial in regulating oxLDL‐induced up‐regulation of ABCA1.

### 3.6 sEH interacts with ABCA1 and decreases ABCA1 phosphorylation

**Figure 6:**
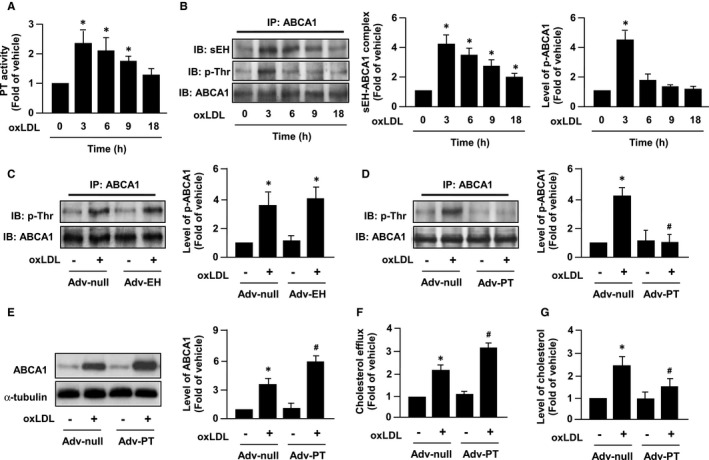
OxLDL promotes the formation sEH‐ABCA1 complex and decreases ABCA1 phosphorylation in macrophages. Macrophages were treated with oxLDL (50 µg/mL) for the indicated times. Cell lysates were assayed for (A) phosphatase (PT) activity and analysed by (B) immunoprecipitation (IP) with normal anti‐ABCA1 antibody. Precipitates were probed for sEH, phosphorylated threonine (p‐Thr) or ABCA1 by immunoblotting (IB). C and D, Macrophages were infected with 50 MOI of adenovirus vector (Adv‐Null) as control, with adenovirus carrying (C) sEH hydrolase domain (Adv‐EH) or (D) PT domain (Adv‐PT) for 24 h, and oxLDL (50 µg/mL) for 3 h. E‐G, Cells were infected with Adv‐null or Adv‐PT for 24 h and then incubated with oxLDL (50 µg/mL) or vehicle control for additional 24 h. E, Expression of ABCA1 and α‐tubulin. F, Cholesterol efflux in the presence of apoAI. G, Intracellular cholesterol. Data are mean ± SD from 5 independent experiments. *P < 0.05 vs time zero or vehicle group, # *P* < 0.05 vs Adv‐null with oxLDL group

Previous studies have reported the phosphorylation status of ABCA1 protein is crucial for its stability.^30^, ^31^, ^32^ We thus determined whether sEH regulates the phosphorylation and turnover of ABCA1 protein in macrophages. We demonstrated that oxLDL time‐dependently increased the activity of phosphatase (Figure 6A). Immunoprecipitation assays revealed that oxLDL increased the interaction of sEH and ABCA1 in a time‐dependent manner, peaking at 3 hours and gradually decreasing to basal levels 18 hours after treatment (Figure 6B). In parallel, oxLDL increased ABCA1 phosphorylation at threonine residues, with maximal effect at 3 hours and decreased thereafter (Figure 6B).

**Figure 7:**
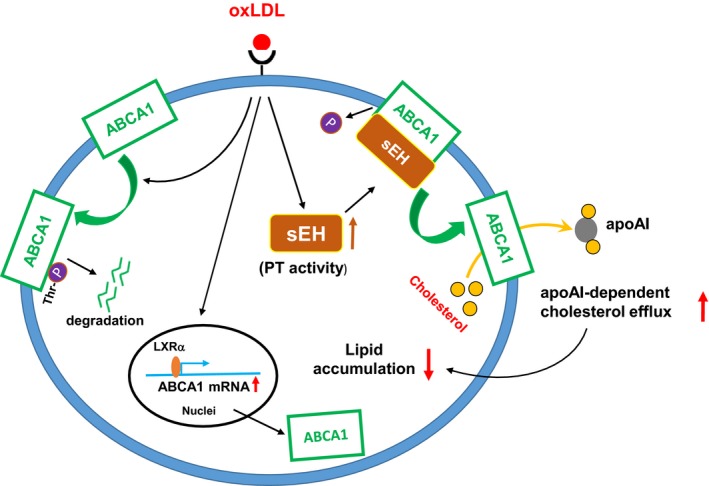
Schematic illustration of proposed mechanism underlying the sEH PT activity‐mediated regulation of ABCA1 expression and cholesterol metabolism in macrophages. As shown, challenge with oxLDL increases sEH PT activity and promotes the formation of sEH‐ABCA1 complex, which in turn decreases ABCA1 phosphorylation at threonine (Thr) residues, leading to the inhibition of ABCA1 degradation and consequently increases cholesterol efflux and decreases lipid accumulation in macrophages

To address whether hydrolase activity or PT activity of sEH is involved in the changes of ABCA1 phosphorylation caused by oxLDL challenge, we amplified the EH activity or PT activity of sEH by overexpressing the PT or EH domains of sEH using an adenovirus system. As shown in Figure 6C,D, overexpression of the PT domain but not of the EH domain abolished the oxLDL‐induced increase in ABCA1 phosphorylation. Moreover, overexpression of the PT domain of sEH augmented the oxLDL‐induced ABCA1 expression and apoAI‐dependent cholesterol efflux, resulting in to a decrease in cholesterol accumulation in macrophage foam cells (Figure 6E‐G). Taken together, these findings suggest that the PT activity of sEH plays an important role in ABCA1 protein expression and its function in cholesterol metabolism of foam cells (Figure 7).

## 4 DISCUSSION

In this study, we identified a novel role of PT activity of sEH in cholesterol homeostasis during the transformation of macrophage foam cells. We first determined that sEH was significantly increased in atherosclerotic aortas and in particular, in intralesional macrophage foam cells. In addition, we found that oxLDL, the most critical atherogenic factor in early atherosclerosis, increased the expression of sEH. These results suggested a role for sEH in the lipid metabolism of macrophage foam cells. Genetic manipulations and pharmacological inhibition revealed that oxLDL‐mediated ABCA1 expression is regulated by the PT activity of sEH. We demonstrated that genetic deletion of sEH or pharmacological inhibition of PT activity, but not inhibition of EH activity, augmented oxLDL‐induced lipid accumulation in macrophages. Moreover, exposure of macrophages to oxLDL increased the physical interaction of sEH with ABCA1 and decreased ABCA1 phosphorylation. More importantly, inhibition of sEH PT activity by ebselen or genetic deletion of sEH prevented the increase in ABCA1 protein expression and cholesterol efflux following oxLDL treatment. By contrast, overexpression of the PT domain of sEH decreased ABCA1 phosphorylation by oxLDL and consequently increased the ABCA1 protein level and cholesterol efflux, leading to the attenuation of lipid accumulation in macrophage foam cells. Together, these results suggest that sEH PT activity has a unique function in regulating cholesterol clearance in macrophage foam cells.

Accumulation of macrophage‐derived foam cells in the intima of the aorta is a critical event for the initiation and progression of atherosclerosis.^16^, ^17^, ^18^ Increase in ABCA1 expression in macrophages promotes cholesterol efflux and decreases cholesterol accumulation of foam cells, thereby retarding the progression of atherosclerosis.^33^, ^34^, ^35^ For instance, overexpression of human ABCA1 in *Apoe* ^−/−^ mice increases cholesterol efflux from macrophages and slows down the progression of atherosclerosis.^36^ Our current findings further confirmed this notion by evidence that overexpression PT domain of sEH increased the ABCA1 protein level and apoAI‐dependent cholesterol efflux, leading to a decrease in intracellular cholesterol of macrophages upon oxLDL challenge. Collectively, we provide the new evidence to support the crucial role of PT activity of sEH in the cholesterol metabolism of foam cells and the development of atherosclerosis.

Under physiological conditions, sEH is an enzyme with the C‐terminal EH activity and N‐terminal PT activity.^1^, ^2^ During the past decade, ample evidence indicates that the EH activity of sEH is involved in the development of cardiovascular diseases, inflammatory diseases and neurodegenerative diseases.^3^, ^4^, ^5^, ^6^, ^7^, ^8^, ^37^ Inhibition of EH activity of sEH results in the accumulation of EETs, which subsequently provide protection against the pathological insults of cardiovascular diseases in vitro and in vivo. The beneficial effects of inhibition of EH activity of sEH are not surprising, because EETs have long been reported to protect against cardiovascular diseases and related complications.^38^, ^39^

Our results showed the inhibition of EH activity by AUDA had no effect on basal level of cholesterol efflux or oxLDL‐induced cholesterol efflux and lipid accumulation in macrophages. These data are inconsistent with those of Shen et al^40^ who found that inhibition of EH activity of sEH increased ABCA1 expression and promoted cholesterol efflux in 3T3L1 adipocytes under normal condition without pro‐obesity stimulation. This protective property may be associated with the regression of atherosclerosis conferred by EH activity inhibitor t‐AUCB in LDL receptor‐deficient mice. On the other hand, we found that inhibition of EH activity by AUDA increased the protein expression of ABCA1 and promoted cholesterol efflux, leading to a decrease in OA‐induced lipid accumulation in Huh7 hepatoma cells (Figure S1). One possible explanation for the discrepancy between our results and those of Shen et al may be due to a difference in cell types or experimental models. Interestingly, our unpublished results showed that combined treatment with EH inhibitor AUDA and EET mixture decreased oxLDL‐induced lipid accumulation in macrophages or further reduced the OA‐induced lipid accumulation in Huh7 hepatoma cells, suggesting the increased level of EETs resulted from the inhibition of EH activity has a protective effect on deregulation of lipid metabolism. Collectively, these findings still suggest the potential therapeutic value of EH activity inhibition for deregulation of lipid metabolism of metabolic diseases.

In contrast to the EH activity, less is known about the biological roles of the PT activity of sEH. Whether PT activity participates in the regulation of cholesterol metabolism and its underlying mechanisms is not fully understood. The PT activity of sEH is assumed to prefer phosphates on lipophilic compounds as substrates.^41^ Despite many studies that have shown that genetic deletion of sEH decreases the pathogenicity of several diseases, both Li et al and Hutchens et al reported that deficiency of sEH in mice exacerbates cardiac dysfunction.^42^, ^43^ As both the PT and EH domains are missing in *EPXH2* ^−/−^ sEH‐deficient mice, it is difficult to determine which domain contributes to the alterations in physiological functions under in vivo conditions. Therefore, we used an in vitro model to isolate the role of PT activity. In our previous studies, we found PT activity of sEH plays a crucial role in vascular endothelial growth factor (VEGF)– and simvastatin‐induced activation of endothelial nitric oxide synthase (eNOS).^44^, ^45^ Inhibiting only the PT activity of sEH augmented VEGF‐ or simvastatin‐induced eNOS phosphorylation and NO production, whereas overexpression of sEH PT domain abrogated eNOS phosphorylation. Consistent with our previous findings, we observed in this study that sEH interacts with ABCA1 and increases ABCA1 stability by decreasing ABCA1 phosphorylation through its PT activity. In view of these data, the PT activity of sEH is likely to increase cholesterol clearance and decrease foam cell formation.

With respect to the function of sEH in cholesterol metabolism, whether EH or PT activity is involved is still not clear. Based on work by EnayetAllah et al^12^ PT activity or EH activity of sEH seems to have opposing roles in regulating cholesterol metabolism in hepatocytes. Overexpression of the PT domain increased the intracellular level of cholesterol, whereas the hydrolase domain decreased it in the human hepatoma cell line HepG2.^12^ Moreover, inhibition of EH activity by pharmacological inhibition to mimic the action of PT activity results in the elevation of cholesterol in HepG2 cells and in livers of mice. However, we did not observe these effects in macrophages without an accompanying oxLDL challenge. In contrast, we found that overexpression of the PT domain but not the EH domain decreased oxLDL‐induced cholesterol accumulation in macrophages. It is possible that the discrepancy between our results and those of EnayetAllah et al may be due to differences in cell types and experimental conditions. Notably, the sEH Glu287Arg variant has lower PT activity and is associated with hyperlipidemia and calcification in CAD patients.^13^, ^14^, ^15^ As of today, the role of PT and EH activity of sEH on cholesterol metabolism has yet to be understood, so further investigations delineating the roles and molecular mechanism underlying the effect of EH and PT activity of sEH on cholesterol metabolism and atherosclerosis are warranted.

The critical role of ABCA1, the major transporter responsible for the apoAI‐dependent cholesterol efflux, in regulating cholesterol homeostasis in macrophages has been well‐characterized.^46^ Increasing ABCA1 expression by clinical and experimental interventions ameliorates lipid accumulation in foam cells and atherosclerosis.^47^, ^48^ ABCA1 protein expression is known to be controlled by transcriptional and post‐transcriptional regulation.^46^ Our results showed that genetic deletion of sEH decreased ABCA1 protein expression without affecting the mRNA expression accompanied by the promoted turnover rate of ABCA1 protein. These results suggest the involvement of post‐transcriptional regulation in the down‐regulation of ABCA1. Moreover, the phosphorylation status of ABCA1 is one of the factors critical for its protein stability.^30^, ^31^, ^32^ Indeed, our co‐IP assays revealed that treatment with oxLDL promoted the interaction between sEH and ABCA1, and regulated the Thr‐phosphorylation of ABCA1. Inhibition of PT activity but not EH activity prevented oxLDL‐induced ABCA1 expression; by contrast, overexpression of the sEH PT domain but not the EH domain diminished oxLDL‐induced Thr‐phosphorylation of ABCA1 and further increased ABCA1 levels in macrophages, consistent with the data of Martinez et al^30^ who reported that phosphorylation of ABCA1 at Thr‐1286 and Thr‐1035 within its PEST domain is critical for calpain‐mediated proteolysis. On the other hand, activation of cAMP/protein kinase A (PKA) signalling increased ABCA1 transporter activity without changing its protein stability by increasing ABCA1 phosphorylation at Ser‐2054 in human cell lines.^31^, ^32^ However, whether the cAMP/protein kinase A (PKA) signalling pathway is involved in sEH‐mediated regulation in ABCA1 expression requires further investigation. Although the detailed mechanism by which PT activity of sEH affects protein stability remains to be determined, here we identify a unique role of sEH in regulating the expression of ABCA1 in macrophages.

Under physiological condition, the lipid homeostasis of whole body is tightly controlled by the cross‐talk between liver, adipose tissues and peripheral tissues including vascular system. Nevertheless, our study contains several limitations because we only examined the role of PT activity of sEH in cholesterol metabolism of macrophage foam cells. From the point in search of drug targets, the role of PT activity of sEH in lipid metabolism should be investigated in more cell types such as hepatocytes and adipocytes. In addition, the role of PT activity of sEH in overall lipid metabolic balance under physiological or pathological conditions should be investigated in vivo. To this end, further investigations describing the implications of PT activity of sEH in lipid homeostasis and metabolic diseases are warranted.

In conclusion, we provide new evidence for PT activity of sEH in the regulation of cholesterol metabolism in macrophage foam cells. The PT activity of sEH reduces oxLDL‐induced ABCA1 phosphorylation and stabilizes ABCA1, which resulted in increased cholesterol efflux and decreased lipid accumulation in macrophage foam cells. Activation of the PT of sEH may be a pharmacological target for atherosclerosis and related cardiovascular diseases.

## CONFLICT OF INTEREST

The authors declare that there are no conflicts of interest.

## AUTHOR CONTRIBUTION

CC Lien, CH Chen, YM Lee, BC Guo, LC Cheng and CC Pan performed the experiments and analysed the data; SK Shyue and TS Lee designed the experiments and wrote the paper.

## Data Availability

The data that support the findings of this study are available on request from the corresponding author. The data are not publicly available due to privacy.
