# Supplementary material for: The phosphatase activity of soluble epoxide hydrolase regulates ATP‐binding cassette transporter‐A1‐dependent cholesterol efflux
Source: J Cell Mol Med. 2019 Aug 22;23(10):6611–21. doi: 10.1111/jcmm.14519 (PMC6787517; doi:10.1111/jcmm.14519)
Supplement: Supplementary file 1 [file JCMM-23-6611-s001.doc]

**Supporting Information**

**The phosphatase activity of soluble epoxide hydrolase regulates ATP-binding cassette transporter-A1-dependent cholesterol efflux**

Chih-Chan Lien, Chia-Hui Chen, Yeng-Ming Lee, Bei-Chia Guo, Li-Ching Cheng, Ching-Chien Pan, Song-Kun Shyue, Tzong-Shyuan Lee

**Supplemental Figure S1.** **Inhibition of EH activity of sEH induces ABCA1-dependent cholesterol efflux and decreases oleic acid (OA)-induced lipid accumulation in Huh7 hepatoma cells**. (A) Huh7 cells were treated with indicated concentrations of AUDA (0, 0.1, 1, 10 μg/ml) for 24 h. Cellular lysates were subjected to western blot analysis for evaluating the protein expression of ABCA1 and α-tubulin. (B) Huh7 cells were treated with AUDA (10 μg/ml) for 12 h, followed by NBD-cholesterol (1 µg/ml) treatment in the presence of AUDA and apoAI (10 µg/ml) for an additional 12 h. Cholesterol efflux was assessed. (C) Cells were pretreated with AUDA (10 μg/ml) for 2 h, followed by OA (100 µg/ml) for an additional 18 h. Intracellular lipid accumulation were evaluated by the Nile red staining. After staining, cellular lysates were analyzed by fluorometry. (D) Representative fluorescent microscopy images of lipid accumulation. Data are mean ± SD from 4 independent experiments. * *P* < 0.05 vs. vehicle group, **^#^** *P* < 0.05 vs. OA-only group.
